# *CFAP43* variant in persistent respiratory symptoms after hematopoietic cell transplantation

**DOI:** 10.1038/s41439-024-00298-5

**Published:** 2024-11-22

**Authors:** Shun Nagasawa, Toyoki Nishimura, Ai Yamada, Sachiyo Kamimura, Masataka Ishimura, Hiroshi Moritake

**Affiliations:** 1 Division of Pediatrics, Faculty of Medicine, University of Miyazaki, Miyazaki, Japan; 2 Department of Pediatrics, Graduate School of Medical Sciences, Kyushu University, Fukuoka, Japan

**Keywords:** Disease genetics, Respiratory tract diseases

## Abstract

We describe a case of *RAS*-associated autoimmune leukoproliferative disease with primary ciliary dyskinesia (PCD)-like symptoms, such as recurrent pneumonia, sinusitis, and otitis media, that occurred 7 years after hematopoietic cell transplantation. Whole-exome sequencing revealed a heterozygous *CFAP43* nonsense variant. Environmental factors related to hematopoietic cell transplantation may have led to PCD symptoms in this patient with this variant. Genetic screening can help avoid subsequent complications during patient management.

## Introduction

Impaired mucociliary clearance of epithelial cilia in the lungs, paranasal sinuses, and ears leads to recurrent and chronic infections of the airways^1,2^. Primary ciliary dyskinesia (PCD) is a heterogeneous disorder of motile cilia dysfunction typically caused by autosomal-recessive disease^3^. Approximately 50 genes have been linked to PCD. More than 70% of patients with PCD have pathogenic variants of these genes^3^.

*CFAP43* encodes a member of the cilia- and flagella-associated protein families. Variants of *CFAP43* have been reported to be responsible for normal pressure hydrocephalus (NPH) and male infertility with multiple morphological abnormalities of the flagella (MMAF)^4,5^. However, the relevance of *CFAP43* variants in PCD is unclear.

Herein, we describe the case of a patient with a heterozygous *CFAP43* variant who developed persistent respiratory symptoms after hematopoietic cell transplantation (HCT).

## Case presentation

An 18-month-old Japanese girl with short stature ( − 2.7 SD) presented with a mass in her left oropharynx. Hepatosplenomegaly was also observed. She was born at term and experienced no respiratory symptoms during the neonatal period. Bronchial asthma was diagnosed during infancy and treated with inhaled corticosteroids and leukotriene antagonists. None of the family members had symptoms mimicking PCD or were diagnosed with PCD, bronchial asthma, or chronic respiratory infection. Blood examinations revealed autoimmune hemolytic anemia and thrombocytopenia. A genetic analysis of mononuclear cells in the peripheral blood revealed a *KRAS* c.37 G > T p.G13C mutation without any mutations in *FAS, FAS-ligand*, or *caspase 10*. No mutation was identified in the buccal mucosal cells, suggesting a somatic mutation. Based on these findings, the patient was diagnosed with *RAS*-associated autoimmune leukoproliferative disease (RALD).

The patient was treated with cyclosporine, mercaptopurine, and prednisolone; however, her symptoms (hepatosplenomegaly and an oropharyngeal mass) and cytopenia persisted. Recurrent otitis media and pneumonia are complications of an immunosuppressive state. The patient was refractory to immunosuppressive therapy; therefore, HCT was conducted. The conditioning regimen was a myeloablative conditioning regimen consisting of busulfan (16.96 mg/kg), fludarabine (120 mg/m^2^), and melphalan (180 mg/m^2^) at 56 months of age. Complete donor chimerism was achieved on Day 43. The tumor in the left oropharynx had disappeared. Furthermore, complete hematological normalization (including a thrombocyte count of >100 × 10^3^/μL) after HCT was successfully achieved on Day 384.

**Fig. 1:**
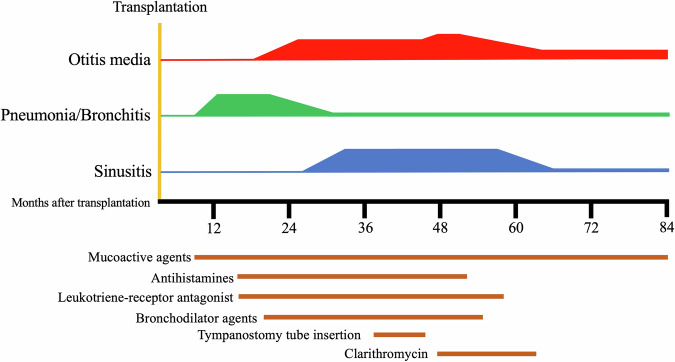
Clinical course of the patient. At 11 months after HCT, the patient’s mild respiratory symptoms were exacerbated, and she developed pneumonia, sinusitis, and otitis media. Mucoactive agents, antihistamines, leukotriene receptor antagonists, bronchodilator agents, and clarithromycin were administered. Tympanostomy tubes were also inserted. At 12 years of age, only mucoactive agents were necessary for chronic otitis media.

**Fig. 2:**
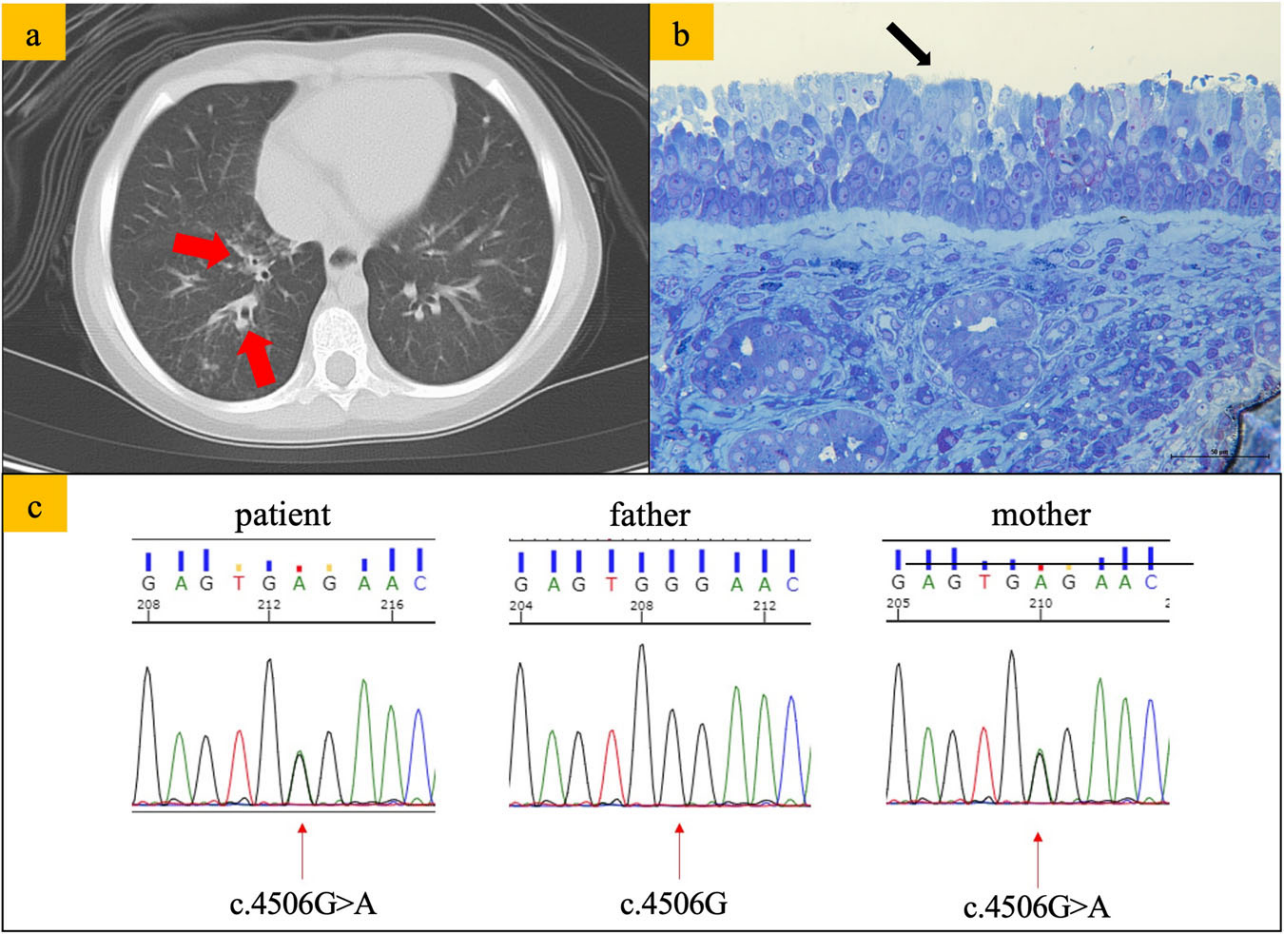
Radiological, pathological, and genetic features of the patient. **a** Computed tomography scan of the chest 23 months after hematopoietic cell transplantation. The arrows highlight changes in bronchial wall thickening in the right lower lobe (red arrows). **b** Toluidine blue staining of the nasal mucosa. On light microscopy of nasal cilia, ciliated cells were only focally observed (black arrow). **c** Electropherograms showing heterozygous variants of *CFAP43* (c.4506 G > A) in the patient and her mother.

Eleven months after HCT, the patient developed pneumonia, sinusitis, and otitis media from mild respiratory symptoms, which required hospitalization with intravenous antibiotics in addition to leukotriene receptor antagonists, antihistamines, mucoactive agents, and bronchodilator agents (Fig. 1). Sputum and otorrhea cultures revealed *Streptococcus pneumoniae*, methicillin-resistant *Staphylococcus aureus*, methicillin-susceptible *Staphylococcus aureus*, and *Haemophilus influenzae*. The administration of several antibiotics to which these pathogens were sensitive succeeded in improving the clinical data, including the WBC and C-reactive protein level. However, the patient’s clinical symptoms did not improve. There was no history or laboratory data suggestive of airway infection (e.g., viral [respiratory syncytial virus, human metapneumovirus, or influenza]), fungal infection, or nontuberculous mycobacteria. Tympanostomy tube insertion was performed in both ears. Immunosuppressive therapy was discontinued 20 months after HCT, although only hypoplasia of the nails associated with mild chronic graft-versus-host disease was recognized. The symptoms related to impaired mucociliary clearance persisted for 7 years, alternating between periods of improvement and exacerbations. Immunological studies revealed complete recovery from the immunocompromised state at 32 months after HCT: IgG, 1384 mg/dL; IgA, 163 mg/dL; IgM, 134 mg/dL; 26.3% CD19-positive lymphocytes; 47.8% CD3-positive lymphocytes; 20.8% CD4-positive lymphocytes; 19.9% CD8-positive lymphocytes; T-cell response to phytohemagglutinin, 37,400 cpm (normal range: 26,000–53,000); and concanavalin A 36,000 cpm (normal range: 20,300–65,700). Chest computed tomography revealed prominent bronchial wall thickening in the lower lobes of the right lung, suggesting impaired ciliary function (Fig. 2a). Light microscopy of a biopsy sample from the nasal mucosa revealed focal ciliated cells (Fig. 2b). Electron microscopy revealed the presence of 1 pair of central singlets and 9 pairs of peripheral doublets with a normal ciliary ultrastructure (data not shown), although the number of cilia was decreased. A targeted next-generation sequencing panel of 32 PCD-related genes (Supplemental Table 1) failed to identify any variants. Whole-exome sequencing of the patient’s peripheral blood mononuclear cells (stored before receiving HCT) and her parents was performed with written informed consent from the patient’s guardians. A heterozygous variant of *CFAP4*3 (c.4506 G > A, p.Trp1502Ter, rs1327299547, minor allele population frequency = 0.000004) was identified in both the patient and her mother (Fig. 2c). We hypothesized that the heterozygous *CFAP4*3 variant might impair ciliary function and cause persistent respiratory symptoms after HCT. Clarithromycin (100 mg/day) was administered for a period of 14 months. After the cessation of clarithromycin, her respiratory function was maintained. At 84 months after HCT, only mucoactive agents were necessary for chronic otitis media.

## Discussion

*CFAP43* is located on chromosome 10. It contains 38 exons encoding a predicted 1665-amino-acid protein that is specifically expressed in the human testis. The biallelic *CFAP43* pathogenic variant causes male infertility with MMAF; however, these patients do not present with any PCD-associated symptoms, such as sinusitis, rhinitis or chronic bronchitis^6^. Morimoto et al. ^4^ described the same heterozygous *CFAP43* variant identified in our patient, which induced NPH in a Japanese family member. Notably, these patients had recurrent respiratory infections and chronic sinusitis. *CFAP43*-deficient mice generated by Morimoto et al. presented a hydrocephalus phenotype with morphological abnormalities in motile cilia, mimicking normal-pressure hydrocephalus in humans^4^. Moreover, immunofluorescence staining of brain ventricular and tracheal tissue using targeted antibodies confirmed the abnormal ciliary protein composition in *CFAP43*-deficient mice. Transmission electron microscopy of respiratory tract cilia from patients (nasal mucosa) and *CFAP43*-deficient mice (tracheal tissue) confirmed the presence of abnormal ciliary ultrastructures. These findings suggest that *CFAP43* affects not only the testis but also other tissues, including epithelial cells of the ventricles of the brain and trachea. However, no other hallmark features of PCD, such as neonatal respiratory distress and chronic ear infections, were identified in patients with a heterozygous *CFAP43* variant. Although our patient experienced recurrent episodes of pneumonia and otitis media during immunosuppressive therapy, no neonatal respiratory distress was observed. These respiratory symptoms worsened after HCT and required long-term treatment. The same heterozygous *CFAP43* variant was found upon sequence analysis of the patient’s mother, who had no respiratory symptoms. This observation suggests that incomplete penetrance of the *CFAP43* variant or environmental modifiers can affect the development of PCD under the *CFAP43* variant. HCT-associated bronchial damage may be associated with PCD mimicking symptoms under the *CFAP43* variant.

Upper respiratory infections are a frequent complication in patients undergoing HCT. Immunosuppression is the main triggering factor, as the airway is the location most exposed to the environment and its microorganisms. The frequency of bacterial rhinosinusitis in patients receiving HCT is much greater (21–37%) than that in immunocompetent patients (5–15%)^7^. Biliary dysfunction and structural damage occur in patients undergoing HCT due to chemotherapy, irradiation, respiratory infections, and graft-versus-host disease. Ortiz et al.^8^ reported reductions in the number of cilia (77%) and goblet cells (50%) and a 50% change in ciliary ultrastructure after HCT. Our patient also presented a decreased number of cilia.

Utimisheva et al.^9^ reported that recovery from sinusitis occurred within 15 days in 5% and within 30 days in 92.5% of patients after HCT. The treatment period exceeded 100 days after HCT in only 2.5% of the patients. The longest treatment period was 240 days. In a previous report, ciliary dysfunction and structural damage after HCT usually improved spontaneously within 10 weeks^1^. Respiratory symptoms persisted for more than 36 months after the patient reached an immunocompetent state. Therefore, the heterozygous *CFAP43* variant may have affected the severe phenotype observed in our patient.

One limitation of our case is that transmission electron microscopy during the recovery period did not reveal abnormal ciliary ultrastructures. We failed to obtain adequate biopsy material when the patient presented with the most severe symptoms. Morimoto et al.^4^ reported a mixture of normal and abnormal ciliary ultrastructures in the nasal mucosa of patients. There is a possibility of abnormal cilia in our patient. More patients are needed to clarify the abnormal ciliary ultrastructure caused by *CFAP43*.

## Conclusion

Heterozygous *CFAP43* nonsense variants may lead to persistent respiratory symptoms after HCT. The establishment of screening for patient management before HCT would help to avoid subsequent complications.

## HGV Database

The relevant data from this Data Report are hosted at the Human Genome Variation Database at 10.6084/m9.figshare.hgv.3450.

## Supplementary information

Supplemental Table 1

## References

[1] Bertrand, B., Collet, S., Eloy, P. & Rombaux, P. Secondary ciliary dyskinesia in upper respiratory tract. *Acta Otorhinolaryngol. Belg.* **54**, 309–316 (2000). 11082767

[2] Rubbo, B. & Lucas, J. S. Clinical care for primary ciliary dyskinesia: current challenges and future directions. *Eur. Respir. Rev.* **26**, 170023 (2017). 28877972 10.1183/16000617.0023-2017 PMC9489029

[3] Dunsky, K., Menezes, M. & Ferkol, T. W. Advances in the Diagnosis and Treatment of Primary Ciliary Dyskinesia: A Review. *JAMA Otolaryngol. Head. Neck Surg.* **147**, 753–759 (2021). 10.1001/jamaoto.2021.0934 34137802

[4] Morimoto, Y. et al. Nonsense mutation in *CFAP43* causes normal-pressure hydrocephalus with ciliary abnormalities. *Neurology* **92**, e2364–e2374 (2019). 31004071 10.1212/WNL.0000000000007505 PMC6598815

[5] Doisaki, S. et al. Somatic mosaicism for oncogenic NRAS mutations in juvenile myelomonocytic leukemia. *Blood* **120**, 1485–1488 (2012). 22753870 10.1182/blood-2012-02-406090

[6] Tang, S. et al. Biallelic mutations in *CFAP43* and *CFAP44* cause male infertility with multiple morphological abnormalities of the sperm flagella. *Am. J. Hum. Genet* **100**, 854–864 (2017). 28552195 10.1016/j.ajhg.2017.04.012 PMC5473723

[7] Ortiz, E., Altemani, A., Vigorito, A. C., Sakano, E. & Nicola, E. M. D. Rhinosinusitis in hematopoietic stem cell-transplanted patients: influence of nasosinus mucosal abnormalities? *Stem Cell Res Ther.* **5**, 133 (2014). 25476934 10.1186/scrt523 PMC4445805

[8] Ortiz, E. et al. Histological features of the nasal mucosa in hematopoietic stem cell transplantation. *Am. J. Rhinol. Allergy* **25**, 191–195 (2011). 29021066 10.2500/ajra.2011.25.3644

[9] Utimisheva, E. S. et al. Incidence, diagnosis and treatment of sinusitis in children and adolescents after hematopoietic stem cell transplantation. *Cell Therapy Transplant.* **8**, 46–53 (2019).

