# Supplementary material for: CFAP43 variant in persistent respiratory symptoms after hematopoietic cell transplantation
Source: Hum Genome Var. 2024 Nov 22;11:41. doi: 10.1038/s41439-024-00298-5 (PMC11582709; doi:10.1038/s41439-024-00298-5)
Supplement: Supplementary file 1 — Supplemental Table 1 [file 41439_2024_298_MOESM1_ESM.docx]

Supplemental table 1.

|  |  |  |  |  |
| --- | --- | --- | --- | --- |
| *ARMC4* | *C21orf59* | *CCDC103* | *CCDC114* | *CCDC151* |
| *CCDC39* | *CCDC40* | *CCDC65* | *CCNO* | *DNAAF1* |
| *DNAAF2* | *DNAAF3* | *DNAAF5* | *DNAH1* | *DNAH11* |
| *DNAH5* | *DNAH8* | *DNAI1* | *DNAI2* | *DNAL1* |
| *DRC1* | *DYX1C1* | *HYDIN* | *LRRC6* | *MCIDAS* |
| *NME8* | *RSPH1* | *RSPH3* | *RSPH4A* | *RSPH9* |
| *SPAG1* | *ZMYND10* |  |  |  |
